# Canadian guidelines for clinical practice: an analysis of their quality and relevance to the care of adults with comorbidity

**DOI:** 10.1186/1471-2296-12-74

**Published:** 2011-07-13

**Authors:** Martin Fortin, Eric Contant, Catherine Savard, Catherine Hudon, Marie-Eve Poitras, José Almirall

**Affiliations:** 1 Department of Family Medicine, Université de Sherbrooke, Sherbrooke, Québec, Canada

## Abstract

**Background:**

Clinical guidelines have been the subject of much criticism in primary care literature partly due to potential conflicts in their implementation among patients with multiple chronic conditions. We assessed the relevance of selected Canadian clinical guidelines on chronic diseases for patients with comorbidity and examined their quality.

**Methods:**

We selected 16 chronic medical conditions according to their frequency of occurrence, complexity of treatment, and pertinence to primary care. Recent Canadian clinical guidelines (2004 - 2009) on these conditions, published in English or French, were retrieved. We assessed guideline relevance to the care of patients with comorbidity with a tool developed by Boyd and colleagues. Quality was assessed using the Appraisal of Guidelines Research and Evaluation (AGREE) instrument.

**Results:**

Regarding relevance, 56.2% of guidelines addressed treatment for patients with multiple chronic conditions and 18.8% addressed the issue for older patients. Fifteen guidelines (93.8%) included specific recommendations for patients with one concurrent condition; only three guidelines (18.8%) addressed specific recommendations for patients with two comorbid conditions and one for more than two concurrent comorbid conditions. Quality of the evaluated guidelines was good to very good in four out of the six domains measured using the AGREE instrument. The domains with lower mean scores were Stakeholder Involvement and Applicability.

**Conclusions:**

The quality of the Canadian guidelines examined is generally good, yet their relevance for patients with two or more chronic conditions is very limited and there is room for improvement in this respect.

## Background

Chronic disease is currently the leading health problem worldwide and represents an important challenge for healthcare systems [1]. The proportion of total direct medical care expenditures devoted to people with chronic diseases has reached $39 billion a year (2004) in Canada [2]. All chronic diseases combined are estimated to account for over 53% of the total economic burden of illness in Canada [2].

The proportion of patients affected by multiple concurrent chronic medical conditions (multimorbidity) is on the rise [3]. Data from different countries demonstrate that this situation results in a high burden for primary care practices where multimorbidity is the rule rather than the exception [3-7]. A Canadian study that included 980 adult patients consulting in a primary care setting found that nine out of 10 patients had more than one chronic condition, and approximately 50% had five or more [4].

Clinical guidelines aim to improve the quality of care provided to patients [8]. Given that guidelines are mostly disease-oriented, they have been the subject of much criticism in primary care literature, partly due to potential conflicting recommendations when implementing those guidelines with patients presenting multiple concurrent chronic conditions [9-12]. This leads to the extended use of physician's own clinical experience and patients' views on treatment choice instead of national guidelines recommendations [13].

A tool recently developed by Boyd and colleagues [14] allowed us to evaluate the applicability of guidelines on chronic diseases for the treatment of subjects with comorbidity (the term comorbidity replaces multimorbidity when the focus is on one specific index disease) [15]. This instrument assesses whether guidelines address treatment for people with several comorbid conditions, as well as patient-centered aspects such as patient preferences and quality of life. Canadian guidelines for chronic diseases have never been evaluated this way. We examined the quality and the relevance of Canadian clinical guidelines for patients with comorbidity for selected chronic diseases.

## Methods

This is a descriptive study based on a literature review and the critical appraisal of clinical guidelines. We initially selected chronic medical conditions according to their prevalence, complexity of treatment, and pertinence to primary care. The selected conditions were: dyslipidemia, chronic impaired renal function, anxiety, heart failure, coagulopathy, obesity, atrial fibrillation, glaucoma, peripheral arterial disease, chronic obstructive pulmonary disease (COPD), asthma, osteoporosis, rheumatoid arthritis, type II diabetes, dementia, high blood pressure, hypothyroidism, and depression. Recent Canadian clinical guidelines (2004 - 2009) published in English or French were selected. Guidelines were identified through the Medline database using the Ovid search engine ("guideline" [publication type] or text word and "Canada" or "Canadian" as text word combined with the corresponding diseases with various spellings as MeSH terms). We also explored several websites known to refer to guidelines: Guidelines Advisory Committee (GAC), Canadian Medical Association InfoBase, Canadian Task Force on Preventive Health Care, Recommended Clinical Practice Guidelines, Canadian Clinical Practice Guideline, Canadian Thoracic Society, Canadian Cardiovascular Society, College of Physicians of Quebec, Canadian Ophthalmological Society and The Canadian Diabetes Association. When more than one guideline had been published by the same organization only the most recent version was retained.

The study was carried out at the Family Medicine Unit of the *Centre de santé et de services sociaux de Chicoutimi *and provided an opportunity for five family medicine residents to complete their mandatory research training under the guidance and supervision of the principal investigator (MF).

To assess the relevance of the guidelines for the care of patients with multiple chronic diseases, we adapted a 14-item tool developed by Boyd and colleagues [14] and expanded it to 17 by adding three items related to medication. This instrument, of relatively recent introduction, is the only one available for this purpose. Boyd et al.'s tool is a checklist in which each item is scored as "yes" or "no". The two principal measurement properties of this instrument of interest to us are its content validity and interrater reliability. Content validity is a qualitative assessment that concerns the extent to which the measure includes all relevant items. It was judged to be adequate by the developers of the tool [14] and by others having used it [15]. We improved its comprehensiveness with the addition of items related to medication. Regarding interrater reliability, the agreement between two reviewers scoring the different items ranged from good to excellent [15]. In this study consensus was reached among independent evaluators. The instrument assesses if guidelines address treatment for older people and for people with several comorbid conditions, as well as patient-centred aspects such as patient preferences and quality of life. A total score was determined for each guideline for comparison purposes.

Guidelines quality was assessed using the Appraisal of Guidelines Research and Evaluation (AGREE) instrument [16]. This instrument comprises 23 items covering six domains. Agreement with the statement for each item is scored on a Likert scale from 1 (complete disagreement) to 4 (complete agreement). This instrument was validated and its reliability is good (Cronbach's alpha 0.64-0.88). A detailed description of the properties of the instrument (reliability and validity) was reported elsewhere [17]. It should be scored by at least two evaluators. Domain scores can be calculated by summing up all the scores obtained for each individual item in a domain and by standardizing the total as a percentage of the maximum possible score for that domain.

The scoring of the two instruments was initially standardized in two steps by five evaluators. First, one guideline (anticoagulant therapy) was discussed and analyzed jointly and consensus was reached for each item. Then, two guidelines (hypertension and anxiety) were read and appraised by the five evaluators independently before results were compared. Differences in rating were resolved by consensus. Thereafter, pairs of evaluators were formed randomly to appraise each guide according to AGREE and Boyd and colleagues' modified instrument. Each evaluator appraised the guidelines independently. A consensus was reached after discussion on divergent items.

## Results

Sixteen Canadian guidelines were selected and appraised [18-33]. Hypothyroidism and depression were removed from the original list since we could not find guidelines for the timeframe of the study (2004 - 2009). Table 1 shows the relevance of Canadian clinical guidelines for the treatment of patients with multiple conditions. Nine guidelines (56.2%) addressed treatment for patients with multiple chronic conditions but only three (18.8%) addressed the issue for older patients. Fifteen guidelines (93.8%) included specific recommendations for patients with one comorbid condition; however, only three guidelines (18.8%) addressed specific recommendations for patients with two comorbid conditions and only one (chronic kidney disease) for more than two concurrent comorbid conditions. All guidelines discussed medication side effects, but only 10 (62.5%) discussed possible medication interactions related to comorbidities. Table 2 shows the performance of each Canadian guideline according to Boyd and colleagues' criteria. The guidelines on chronic impaired renal function, heart failure, diabetes mellitus and dementia had the best performance scores, whereas the guidelines on atrial fibrillation and rheumatoid arthritis had the lowest.

**Table 1:** Relevance of Canadian clinical guidelines for the treatment of patients with multiple conditions.

	No. of guidelines (%)
Issues addressed	
Guideline addressed treatment for older patients	8 (50)
Guideline addressed treatment for patients with multiple comorbid conditions	9 (56.2)
Guideline addressed treatment for older patients with multiple comorbid conditions	3 (18.8)
Quality of evidence	
Quality of evidence discussed for older patients	9 (56.2)
Quality of evidence discussed for patients with multiple comorbid conditions	12 (75.0)
Quality of evidence discussed for older patients with comorbid conditions	3 (18.8)
Recommendations	
Specific recommendations for patients with one comorbid condition	15 (93.8)
Specific recommendations for patients with two comorbid conditions	3 (18.8)
Specific recommendations for patients with more than two comorbid conditions	1 (6.2)
Burden of treatment	
Time needed to treat to benefit from treatment in the context of life expectancy discussed	9 (56.2)
Guideline discussed burden of comprehensive treatment on patients or caregivers	6 (37.5)
Guideline discussed patients' financial burden	4 (25)
Guideline discussed patients' quality of life	13 (81.2)
Patient preferences	
Guideline discussed patients' preferences	9 (56.2)
Medication	
Guideline discussed medications' side effects	16 (100)
Guideline is adapted to possible medications' side effects	12 (75)
Guideline discussed possible medications' interactions related to comorbidities	10 (62.5)

**Table 2:** Guidelines performance according to Boyd and colleagues' modified criteria.

	Number of criteria satisfied (%)
Dyslipidemia,[18]	8 (47)
Chronic impaired renal function[19]	11 (65)
Heart failure[20]	13 (76)
Anticoagulant therapy[21]	7 (41)
Obesity[22]	7 (41)
Atrial fibrillation[23]	6 (35)
Peripheral arterial disease[24]	9 (53)
COPD[25]	9 (53)
Osteoporosis[26]	9 (53)
Rheumatoid arthritis	5 (29)
Diabetes mellitus[28]	15 (88)
Asthma[29]	6 (35)
Dementia[30]	12 (71)
Glaucoma[31]	9 (53)
Anxiety[32]	8 (47)
Hypertension[33]	7 (41)

The individual standardized AGREE domain scores and mean scores are shown in Table 3. Scope and Purpose and Clarity and Presentation obtained the highest and second highest mean scores, respectively. Rigor of Development and Editorial Independence were good although the latter showed much variability. The Stakeholder Involvement and Applicability domains obtained the lowest scores. Five guidelines showed good quality (score above 60%) in all domains: Chronic Impaired Renal Function, Obesity, COPD, Diabetes Mellitus and Glaucoma, the last two getting the highest marks.

**Table 3:** Individual standardized AGREE domain scores (in %) for the guidelines studied.

	Scope and purpose	Stakeholder involvement	Rigor of development	Clarity and presentation	Applicability	Editorial independence
Dyslipidemia[18]	83.3	33.3	81.0	100	44.4	100
Chronic impaired renal function[19]	100	66.6	78.6	100	72.2	100
Heart failure[20]	77.8	62.5	71.4	87.5	38.8	100
Anticoagulant therapy[21]	100	62.5	42.9	100	75	62.3
Obesity[22]	100	62.5	100	91.7	83.3	91.7
Atrial fibrillation[23]	88.9	25.0	64.3	71.0	22.2	25.0
Peripheral arterial disease[24]	94.4	25.3	78.6	100	33.3	50.0
COPD[25]	94.4	75.0	78.6	100	66.7	66.7
Osteoporosis[26]	100	25.0	71.4	75.0	33.3	33.3
Rheumatoid arthritis	88.9	25.0	47.6	58.3	22.2	41.7
Diabetes mellitus[28]	88.9	83.3	100	100	88.9	100
Asthma[29]	88.9	66.7	85.7	91.7	16.7	66.7
Dementia[30]	100	79.2	90.5	58.3	11.1	100
Glaucoma[31]	100	66.7	100	100	77.8	100
Anxiety[32]	91.7	68.3	85.6	100	33.3	66.7
Hypertension[33]	91.7	55.0	97.1	95.0	48.9	86.7

## Discussion

Although 56.2% of the guidelines addressed the treatment of patients with multiple comorbid conditions, only a minority (18.8%) provided specific treatment recommendations for patients with two other conditions and even less for those with more than two comorbid conditions. This does not reflect the reality of primary care where there is a very high prevalence of multimorbidity [3-5]. These results are similar to an Australian study reporting that 53% of 17 guidelines addressed treatment for patients with multiple comorbid conditions [15]. One can argue that due to the diversity of chronic diseases seen in primary care, one cannot account for all possible combinations and adjust guidelines accordingly. Chances are that the evidence to support such recommendations would be weak or absent as randomized control trials often exclude patients with comorbidity or fail to describe the morbidity burden of their participants in published reports [34]. Of particular concern is the fact that the issue of multiple comorbid conditions is rarely discussed for older patients in the Canadian guidelines despite the strong association between aging and the presence of multiple chronic conditions [3-5]. This situation is not surprising considering the reliance on randomized control trials (RCTs) that have excluded patients with comorbidities in the development of evidence-based guidelines [34]. This calls for a dramatic shift in clinical research towards more pragmatic trials to generate evidence and a greater implication of the primary care sector in the research process. Research on new models of care for more complex patients and implying interdisciplinary collaboration is promising but thus far, publications are scarce [35]. A relatively low coverage of burden of treatment and patient preferences is also an indication of the lack of focus on the patient's perspective in Canadian guidelines on chronic diseases. This is in line with the disease-oriented paradigm from which the guidelines arise. This is a concrete example of the misconceptualization of the primary care role from the guidelines developer perspective as reported by Starfield [9]. All guidelines discussed medication side effects, with 62.5% of them discussing, to some extent, possible interactions related to comorbidity. However, in most cases, the discussion was superficial and limited to a few warnings. Both drug-drug and drug-disease interactions are possible in patients with comorbidities [36]. Appropriate management of comorbidity should be included in guidelines for particular diseases [37]. Most guidelines failed to discuss the burden of comprehensive treatment on patients or caregivers. This is especially of concern for older patients with multimorbidity who may find it very difficult to adopt the regimen issued from the many guidelines that would apply to them [14]. Summing up the score for individual items for each guideline from Boyd and colleagues' instrument resulted in an overall score allowing for comparisons between guidelines. Guidelines for diabetes mellitus performed the best. This is reassuring as diabetes is associated with many chronic diseases and complications. However, a low score for atrial fibrillation is especially of concern, as the treatment of this condition will interfere with many other conditions.

The quality of the guidelines evaluated in this study was good to very good in four out of the six domains measured by the AGREE instrument. This evaluation is necessary to ensure that the guidelines are of good quality according to Boyd and colleagues' criteria. The domains with lower scores were: Stakeholder Involvement and Applicability. Guidelines devoted to improved quality of primary care would certainly benefit from a strong partnership with primary care providers in charge of applying the guidelines and this is especially of concern due to the high prevalence of chronic diseases in this setting. The low scores of those two criteria call for a more pragmatic way of elaborating and formulating guidelines in order to better fit the needs of targeted users. There is no use for a guideline if its implementation into practice is not facilitated and does not entail a shift of paradigm from the more disease-oriented guideline developers to the more patient-oriented users [38,39]. The Scope and Purpose, and Clarity and Presentation domains obtained very good scores; this, along with the good scores of the Rigor of Development domain, speaks well to the scientific quality and validity of guidelines that is the hallmark of Canadian research.

This study has limitations. Although our selection criteria allowed us to include a variety of important medical conditions, our results cannot be generalized to other Canadian guidelines not included in this study. Another limitation is that Boyd and colleagues' instrument is a relatively new tool; this may raise questions about its use in evaluating guidelines. The AGREE instrument provides a well validated framework for the assessment of the quality of guidelines. However, ratings can be influenced by the reviewer's background knowledge of the guideline subject. We tried to reduce this bias by distributing guidelines randomly among the reviewers.

## Conclusion

Our results show that the quality of Canadian guidelines is good, particularly in their scope and purpose, as well as in clarity and presentation. However, their relevance for patients with more than two comorbid conditions is very limited and there is room for improvement in this respect. It may be the reflection of some disconnection between guideline developers and primary care providers. Clinical judgment is still necessary to adjust guideline recommendations for patients with comorbidity.

## Competing interests

The authors declare that they have no competing interests.

## Authors' contributions

MF and M-EP conceived and designed the study. They also supervised the data collection, analyzed the data and drafted the manuscript. EC and CS participated in the data collection and analysis and critically reviewed the manuscript. JA and CH participated in the data analysis, and helped draft the manuscript. All authors gave their final approval of the version of the manuscript submitted for publication. MF takes responsibility for the integrity of the work as a whole.

## Pre-publication history

The pre-publication history for this paper can be accessed here:

http://www.biomedcentral.com/1471-2296/12/74/prepub
